# (Adamantan-1-yl)(phenyl­sulfan­yl)methanone

**DOI:** 10.1107/S1600536812026116

**Published:** 2012-06-16

**Authors:** Adel S. El-Azab, Alaa A.-M. Abdel-Aziz, Ibrahim A. Al-Swaidan, Seik Weng Ng, Edward R. T. Tiekink

**Affiliations:** a Department of Pharmaceutical Chemistry, College of Pharmacy, King Saud University, Riyadh 11451, Saudi Arabia; b Department of Organic Chemistry, Faculty of Pharmacy, Al-Azhar University, Cairo 11884, Egypt; c Department of Medicinal Chemistry, Faculty of Pharmacy, University of Mansoura, Mansoura 35516, Egypt; d Department of Chemistry, University of Malaya, 50603 Kuala Lumpur, Malaysia; e Chemistry Department, Faculty of Science, King Abdulaziz University, PO Box 80203 Jeddah, Saudi Arabia

## Abstract

Two independent mol­ecules (*A* and *B*) comprises the asymmetric unit of the title ester, C_17_H_20_OS. The phenyl ring is inclined with respect to the thio­carboxyl group, forming dihedral angles of 58.95 (6) (in mol­ecule *A*) and 62.28 (6)° (in mol­ecule *B*). In each independent mol­ecule, one adamantyl methyl­ene C atom is nearly coplanar with the thio­carboxyl group. The major difference between mol­ecules *A* and *B* relates to the relationship between the S atom and the coplanar adamantyl methyl­ene C atom [C_a_—C_q_—C_c_—S torsion angles = 178.25 (8) and 6.81 (13)°, respectively; C_a_ = adamantyl methyl­ene C atom, C_q_ = quaternary C atom and C_c_ = carbonyl C atom], whereby the S atom in mol­ecule *A* has an *anti* relationship with the methyl­ene C atom and in mol­ecule *B*, the S atom is *syn*. In the crystal, C—H⋯π inter­actions are formed leading to supra­molecular layers in the *ac* plane.

## Related literature

For applications of thio­esters in organic synthesis, see: Shah *et al.* (2002 ▶); Manabe *et al.* (2007 ▶); Horst *et al.* (2007 ▶). For the synthesis, see: El-Azab & Abdel-Aziz *et al.* (2012 ▶).
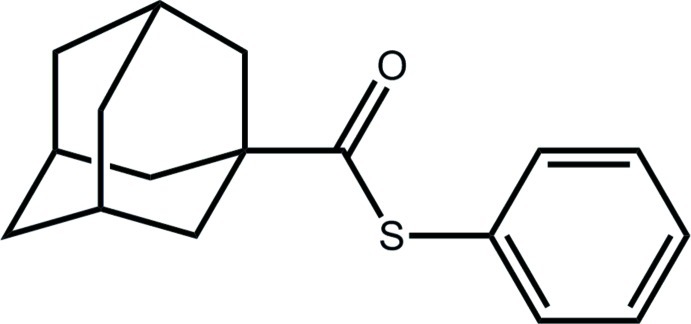


## Experimental

### 

#### Crystal data

C_17_H_20_OS
*M* *_r_* = 272.39
Monoclinic, 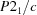

*a* = 6.3545 (1) Å
*b* = 39.4559 (5) Å
*c* = 11.3878 (1) Å
β = 99.879 (1)°
*V* = 2812.84 (6) Å^3^
*Z* = 8
Cu *K*α radiation
μ = 1.94 mm^−1^
*T* = 100 K
0.30 × 0.25 × 0.20 mm

#### Data collection

Agilent SuperNova Dual diffractometer with an Atlas detector
Absorption correction: multi-scan (*CrysAlis PRO*; Agilent, 2012 ▶) *T*_min_ = 0.881, *T*_max_ = 1.000
11270 measured reflections
5753 independent reflections
5445 reflections with *I* > 2σ(*I*)
*R*_int_ = 0.015

#### Refinement

*R*[*F* ^2^ > 2σ(*F* ^2^)] = 0.033
*wR*(*F* ^2^) = 0.087
*S* = 1.02
5753 reflections
343 parameters
H-atom parameters constrained
Δρ_max_ = 0.30 e Å^−3^
Δρ_min_ = −0.40 e Å^−3^

### 

Data collection: *CrysAlis PRO* (Agilent, 2012 ▶); cell refinement: *CrysAlis PRO*; data reduction: *CrysAlis PRO*; program(s) used to solve structure: *SHELXS97* (Sheldrick, 2008 ▶); program(s) used to refine structure: *SHELXL97* (Sheldrick, 2008 ▶); molecular graphics: *ORTEP-3* (Farrugia, 1997 ▶), *DIAMOND* (Brandenburg, 2006 ▶) and *QMol* (Gans & Shalloway, 2001 ▶); software used to prepare material for publication: *publCIF* (Westrip, 2010 ▶).

## Supplementary Material

Crystal structure: contains datablock(s) global, I. DOI: 10.1107/S1600536812026116/xu5558sup1.cif

Structure factors: contains datablock(s) I. DOI: 10.1107/S1600536812026116/xu5558Isup2.hkl

Supplementary material file. DOI: 10.1107/S1600536812026116/xu5558Isup3.cml

Additional supplementary materials:  crystallographic information; 3D view; checkCIF report

## Figures and Tables

**Table 1:** Hydrogen-bond geometry (Å, °) *Cg*1 and *Cg*2 are the centroids of the C12–C17 and C29–C34 benzene rings, respectively.

*D*—H⋯*A*	*D*—H	H⋯*A*	*D*⋯*A*	*D*—H⋯*A*
C22—H22⋯*Cg*1^i^	1.00	2.81	3.6129 (13)	138
C34—H34⋯*Cg*1	0.95	2.73	3.4234 (14)	131
C15—H15⋯*Cg*2^ii^	0.95	2.86	3.6668 (16)	143

Symmetry codes: (i) 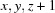; (ii) 
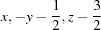
.

**Fig. 1.**
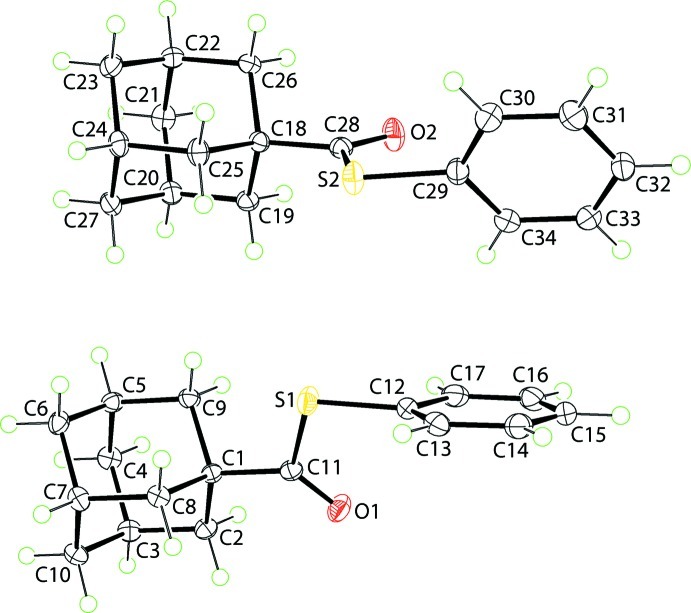
The molecular structure of (I) showing the atom-labelling scheme and displacement ellipsoids at the 50% probability level.

**Fig. 2.**
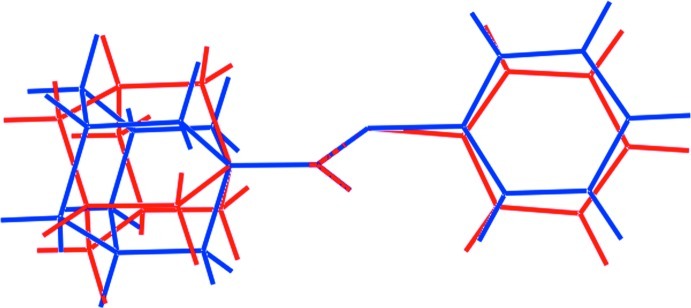
Superimposition of the S1-containing molecule (red) and the inverted S2-containing molecule (blue) in (I). The COS moieties have been superimposed.

**Fig. 3.**
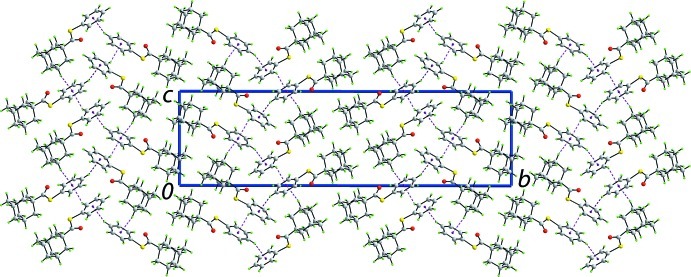
A view in projection down the a axis of the unit-cell contents for (I). The C—H···π interactions are shown as purple dashed lines.

## supplementary crystallographic information

### Comment

It is widely known that thioesters are useful building blocks for organic transformations, for example thioesters are important in many areas of organic chemistry, particularly in peptide, protein, and β-lactam synthesis (Shah *et al.*, 2002). Furthermore, they find application in peptide bond formation (Manabe *et al.*, 2007) and in natural product synthesis (Horst *et al.*, 2007). The title compound, *S*-phenyl adamantane-1-carbothioate (I) was synthesized according to El-Azab & Abdel-Aziz (2012) and herein, we describe its crystal structure determination.

Two independent molecules comprise the asymmetric unit of (I), Fig. 1. As seen from the overlay diagram, Fig. 2, there are non-trivial differences between the molecules when the S1-containing molecule is superimposed with the inverted S2-containing molecule. The dihedral angle between the plane through the COS atoms and the *S*-bound phenyl ring is 58.95 (6)° for the S1-containing molecule and 62.28 (6)° for the S2-containing molecule. There is a more dramatic difference in the relative orientations between the COS residue and the adamantyl group. This is best quantified in the values of the C2—C1—C11—S1 and C25—C18—C28—S2 torsion angles of 178.25 (8) and 6.81 (13)°, respectively, *i.e*. where there is an almost co-planar relationship between the S and one methylene-C atom. The difference arises in the the S1 atom has *anti* relationship with the co-planar methylene-C atom and the S2 atom has a *syn* relationship.

In the crystal packing, C—H···π interactions are formed with the C12—C17 ring forming two such interactions, Table 1. The result is the formation of a supramolecular layer in the *ac* plane with the adamantyl groups inter-digitating along the *b* axis, Fig. 2.

### Experimental

Trifluoroacetic acid (0.4 equiv.) was added drop-wise to a stirred solution of 1-adamantane carboxylic acid (1 equiv.) and thiophenol (1 equiv) in dry CH_3_CN (0.01 mol/*L*) over a period of 15 min. at room temperature. After being stirred for 5 h at 333 K, the mixture was quenched by adding ammonium chloride solution (5 ml), extracted with ethylacetate, washed with brine and dried over anhydrous Na_2_SO_4_. The product, obtained after evaporation of the solvent, was purified by column chromatography using mixture of hexane and CHCl_3_ as eluent. The crystals were obtained by slow evaporation of the eluent. *M*.pt; 341 K; 86% yield. IR (KBr): 1680 cm^-1^ (C═O). ^1^H NMR (CDCl_3_): δ 7.42–7.36 (m, 5H), 2.13–2.11 (m, 3H), 2.04–2.02 (m, 6H), 1.82–1.76 (m, 6H). ^13^C NMR (CDCl_3_): δ 28.1, 28.3, 36.4, 39.3, 49.1, 128.0, 129.1, 134.8, 135.1, 204.2.

### Refinement

Carbon-bound H-atoms were placed in calculated positions [C—H = 0.95 to 1.00 Å, *U*_iso_(H) = 1.2*U*_eq_(C)] and were included in the refinement in the riding model approximation.

### Crystal data

**Table:**

C_17_H_20_OS	*F*(000) = 1168
*M**_r_* = 272.39	*D*_x_ = 1.286 Mg m^−^^3^
Monoclinic, *P*2_1_/*c*	Cu *K*α radiation, λ = 1.54184 Å
Hall symbol: -P 2ybc	Cell parameters from 7141 reflections
*a* = 6.3545 (1) Å	θ = 4.5–76.4°
*b* = 39.4559 (5) Å	µ = 1.94 mm^−^^1^
*c* = 11.3878 (1) Å	*T* = 100 K
β = 99.879 (1)°	Prism, colourless
*V* = 2812.84 (6) Å^3^	0.30 × 0.25 × 0.20 mm
*Z* = 8	

### Data collection

**Table:**

Agilent SuperNova Dual diffractometer with an Atlas detector	5753 independent reflections
Radiation source: SuperNova (Cu) X-ray Source	5445 reflections with *I* > 2σ(*I*)
Mirror monochromator	*R*_int_ = 0.015
Detector resolution: 10.4041 pixels mm^-1^	θ_max_ = 76.6°, θ_min_ = 4.5°
ω scan	*h* = −7→7
Absorption correction: multi-scan (*CrysAlis PRO*; Agilent, 2012)	*k* = −47→48
*T*_min_ = 0.881, *T*_max_ = 1.000	*l* = −8→14
11270 measured reflections	

### Refinement

**Table:**

Refinement on *F*^2^	Primary atom site location: structure-invariant direct methods
Least-squares matrix: full	Secondary atom site location: difference Fourier map
*R*[*F*^2^ > 2σ(*F*^2^)] = 0.033	Hydrogen site location: inferred from neighbouring sites
*wR*(*F*^2^) = 0.087	H-atom parameters constrained
*S* = 1.02	*w* = 1/[σ^2^(*F*_o_^2^) + (0.0484*P*)^2^ + 1.0716*P*] where *P* = (*F*_o_^2^ + 2*F*_c_^2^)/3
5753 reflections	(Δ/σ)_max_ = 0.001
343 parameters	Δρ_max_ = 0.30 e Å^−^^3^
0 restraints	Δρ_min_ = −0.40 e Å^−^^3^

### Fractional atomic coordinates and isotropic or equivalent isotropic displacement parameters (Å^2^)

**Table:**

	*x*	*y*	*z*	*U*_iso_*/*U*_eq_	
S1	0.46660 (5)	0.389463 (8)	0.15443 (3)	0.02399 (9)	
S2	0.08761 (5)	0.329812 (7)	0.36140 (3)	0.02206 (9)	
O1	0.76535 (15)	0.39786 (2)	0.02056 (8)	0.0241 (2)	
O2	0.44170 (15)	0.29759 (2)	0.45497 (8)	0.0251 (2)	
C1	0.76163 (18)	0.44208 (3)	0.16696 (10)	0.0137 (2)	
C2	0.95311 (18)	0.45667 (3)	0.11779 (10)	0.0166 (2)	
H2A	1.0706	0.4399	0.1275	0.020*	
H2B	0.9109	0.4616	0.0317	0.020*	
C3	1.02995 (18)	0.48936 (3)	0.18489 (10)	0.0171 (2)	
H3	1.1546	0.4987	0.1525	0.021*	
C4	1.09809 (19)	0.48149 (3)	0.31807 (11)	0.0190 (2)	
H4A	1.2160	0.4648	0.3291	0.023*	
H4B	1.1499	0.5024	0.3616	0.023*	
C5	0.90662 (19)	0.46717 (3)	0.36777 (10)	0.0174 (2)	
H5	0.9507	0.4621	0.4546	0.021*	
C6	0.72444 (19)	0.49318 (3)	0.35115 (10)	0.0179 (2)	
H6A	0.6010	0.4840	0.3834	0.021*	
H6B	0.7723	0.5142	0.3953	0.021*	
C7	0.65749 (18)	0.50116 (3)	0.21815 (11)	0.0168 (2)	
H7	0.5391	0.5182	0.2075	0.020*	
C8	0.58022 (18)	0.46848 (3)	0.15095 (10)	0.0152 (2)	
H8A	0.5350	0.4735	0.0652	0.018*	
H8B	0.4557	0.4593	0.1820	0.018*	
C9	0.82946 (19)	0.43447 (3)	0.30135 (10)	0.0162 (2)	
H9A	0.7068	0.4250	0.3337	0.019*	
H9B	0.9456	0.4174	0.3129	0.019*	
C10	0.84922 (19)	0.51560 (3)	0.16903 (11)	0.0182 (2)	
H10A	0.8063	0.5211	0.0835	0.022*	
H10B	0.8992	0.5367	0.2121	0.022*	
C11	0.68647 (18)	0.40943 (3)	0.10050 (10)	0.0157 (2)	
C12	0.4232 (2)	0.35256 (3)	0.06489 (10)	0.0186 (2)	
C13	0.2217 (2)	0.34757 (3)	−0.00245 (11)	0.0219 (3)	
H13	0.1133	0.3642	−0.0035	0.026*	
C14	0.1807 (2)	0.31787 (4)	−0.06838 (12)	0.0280 (3)	
H14	0.0429	0.3141	−0.1140	0.034*	
C15	0.3390 (3)	0.29386 (3)	−0.06799 (12)	0.0310 (3)	
H15	0.3103	0.2737	−0.1138	0.037*	
C16	0.5399 (3)	0.29911 (3)	−0.00078 (12)	0.0298 (3)	
H16	0.6485	0.2826	−0.0010	0.036*	
C17	0.5832 (2)	0.32837 (3)	0.06684 (11)	0.0232 (3)	
H17	0.7201	0.3318	0.1138	0.028*	
C18	0.41351 (18)	0.35295 (3)	0.54008 (10)	0.0144 (2)	
C19	0.61791 (19)	0.36792 (3)	0.50444 (10)	0.0169 (2)	
H19A	0.7273	0.3500	0.5068	0.020*	
H19B	0.5855	0.3769	0.4221	0.020*	
C20	0.70394 (19)	0.39656 (3)	0.59103 (11)	0.0183 (2)	
H20	0.8358	0.4063	0.5674	0.022*	
C21	0.7582 (2)	0.38215 (3)	0.71780 (11)	0.0211 (3)	
H21A	0.8683	0.3643	0.7206	0.025*	
H21B	0.8163	0.4003	0.7740	0.025*	
C22	0.5563 (2)	0.36727 (3)	0.75447 (10)	0.0197 (2)	
H22	0.5920	0.3579	0.8370	0.024*	
C23	0.3884 (2)	0.39527 (3)	0.75166 (11)	0.0198 (2)	
H23A	0.2581	0.3859	0.7763	0.024*	
H23B	0.4446	0.4135	0.8080	0.024*	
C24	0.33386 (19)	0.40958 (3)	0.62496 (11)	0.0174 (2)	
H24	0.2234	0.4277	0.6227	0.021*	
C25	0.24680 (19)	0.38120 (3)	0.53755 (11)	0.0183 (2)	
H25A	0.1146	0.3718	0.5597	0.022*	
H25B	0.2110	0.3906	0.4559	0.022*	
C26	0.4675 (2)	0.33886 (3)	0.66795 (10)	0.0180 (2)	
H26A	0.3372	0.3292	0.6918	0.022*	
H26B	0.5745	0.3205	0.6710	0.022*	
C27	0.5349 (2)	0.42451 (3)	0.58783 (11)	0.0189 (2)	
H27A	0.5915	0.4430	0.6429	0.023*	
H27B	0.4996	0.4340	0.5064	0.023*	
C28	0.33992 (19)	0.32324 (3)	0.45677 (10)	0.0164 (2)	
C29	0.0397 (2)	0.29028 (3)	0.28765 (11)	0.0189 (2)	
C30	−0.1416 (2)	0.27231 (3)	0.30447 (11)	0.0228 (3)	
H30	−0.2272	0.2802	0.3595	0.027*	
C31	−0.1958 (2)	0.24277 (3)	0.23994 (12)	0.0257 (3)	
H31	−0.3202	0.2306	0.2502	0.031*	
C32	−0.0694 (2)	0.23103 (3)	0.16072 (11)	0.0247 (3)	
H32	−0.1073	0.2108	0.1169	0.030*	
C33	0.1126 (2)	0.24884 (3)	0.14535 (11)	0.0232 (3)	
H33	0.2001	0.2406	0.0919	0.028*	
C34	0.1671 (2)	0.27874 (3)	0.20787 (11)	0.0213 (3)	
H34	0.2899	0.2912	0.1963	0.026*	

### Atomic displacement parameters (Å^2^)

**Table:**

	*U*^11^	*U*^22^	*U*^33^	*U*^12^	*U*^13^	*U*^23^
S1	0.02653 (17)	0.02207 (16)	0.02655 (16)	−0.00994 (12)	0.01353 (13)	−0.01064 (12)
S2	0.02095 (16)	0.01698 (15)	0.02578 (16)	0.00343 (11)	−0.00291 (12)	−0.00611 (11)
O1	0.0261 (5)	0.0264 (5)	0.0218 (4)	−0.0040 (4)	0.0103 (4)	−0.0081 (4)
O2	0.0249 (5)	0.0175 (4)	0.0313 (5)	0.0064 (4)	0.0003 (4)	−0.0048 (4)
C1	0.0128 (5)	0.0158 (5)	0.0128 (5)	−0.0002 (4)	0.0028 (4)	0.0004 (4)
C2	0.0142 (5)	0.0201 (6)	0.0166 (5)	−0.0003 (4)	0.0056 (4)	−0.0003 (4)
C3	0.0122 (5)	0.0201 (6)	0.0197 (6)	−0.0026 (4)	0.0049 (4)	0.0011 (4)
C4	0.0140 (5)	0.0217 (6)	0.0200 (6)	−0.0027 (4)	−0.0006 (4)	−0.0001 (5)
C5	0.0187 (6)	0.0194 (6)	0.0134 (5)	−0.0034 (5)	0.0012 (4)	0.0003 (4)
C6	0.0182 (6)	0.0177 (6)	0.0188 (6)	−0.0042 (4)	0.0063 (4)	−0.0043 (4)
C7	0.0135 (5)	0.0152 (5)	0.0222 (6)	0.0003 (4)	0.0039 (4)	0.0001 (4)
C8	0.0118 (5)	0.0166 (5)	0.0170 (5)	0.0000 (4)	0.0019 (4)	0.0010 (4)
C9	0.0181 (6)	0.0158 (5)	0.0143 (5)	−0.0007 (4)	0.0011 (4)	0.0017 (4)
C10	0.0177 (6)	0.0166 (5)	0.0208 (6)	−0.0016 (4)	0.0043 (4)	0.0031 (4)
C11	0.0150 (5)	0.0176 (5)	0.0141 (5)	0.0010 (4)	0.0015 (4)	0.0011 (4)
C12	0.0259 (6)	0.0151 (5)	0.0156 (5)	−0.0034 (5)	0.0056 (5)	−0.0006 (4)
C13	0.0259 (7)	0.0192 (6)	0.0207 (6)	−0.0040 (5)	0.0047 (5)	0.0023 (5)
C14	0.0381 (8)	0.0264 (7)	0.0185 (6)	−0.0132 (6)	0.0022 (5)	−0.0001 (5)
C15	0.0594 (10)	0.0170 (6)	0.0189 (6)	−0.0095 (6)	0.0129 (6)	−0.0028 (5)
C16	0.0490 (9)	0.0176 (6)	0.0266 (7)	0.0070 (6)	0.0172 (6)	0.0043 (5)
C17	0.0282 (7)	0.0225 (6)	0.0193 (6)	0.0025 (5)	0.0051 (5)	0.0048 (5)
C18	0.0143 (5)	0.0132 (5)	0.0163 (5)	0.0014 (4)	0.0045 (4)	0.0008 (4)
C19	0.0172 (6)	0.0164 (5)	0.0188 (5)	0.0003 (4)	0.0082 (4)	0.0011 (4)
C20	0.0148 (6)	0.0181 (6)	0.0235 (6)	−0.0023 (4)	0.0077 (4)	−0.0009 (5)
C21	0.0165 (6)	0.0226 (6)	0.0230 (6)	0.0021 (5)	−0.0005 (5)	−0.0026 (5)
C22	0.0258 (6)	0.0191 (6)	0.0142 (5)	0.0009 (5)	0.0036 (5)	0.0022 (4)
C23	0.0211 (6)	0.0202 (6)	0.0202 (6)	−0.0018 (5)	0.0094 (5)	−0.0034 (5)
C24	0.0154 (6)	0.0144 (5)	0.0227 (6)	0.0022 (4)	0.0040 (4)	−0.0023 (4)
C25	0.0152 (6)	0.0162 (5)	0.0226 (6)	0.0033 (4)	0.0012 (4)	−0.0025 (4)
C26	0.0228 (6)	0.0152 (5)	0.0171 (5)	0.0007 (4)	0.0061 (5)	0.0032 (4)
C27	0.0219 (6)	0.0135 (5)	0.0222 (6)	−0.0010 (4)	0.0064 (5)	0.0001 (4)
C28	0.0166 (6)	0.0163 (5)	0.0173 (5)	0.0006 (4)	0.0056 (4)	0.0015 (4)
C29	0.0226 (6)	0.0155 (5)	0.0173 (5)	0.0011 (5)	−0.0002 (4)	−0.0013 (4)
C30	0.0231 (6)	0.0239 (6)	0.0216 (6)	0.0000 (5)	0.0042 (5)	−0.0021 (5)
C31	0.0266 (7)	0.0236 (6)	0.0271 (7)	−0.0061 (5)	0.0051 (5)	−0.0014 (5)
C32	0.0355 (7)	0.0171 (6)	0.0204 (6)	−0.0037 (5)	0.0019 (5)	−0.0023 (5)
C33	0.0338 (7)	0.0190 (6)	0.0180 (6)	0.0010 (5)	0.0078 (5)	0.0000 (5)
C34	0.0261 (6)	0.0181 (6)	0.0203 (6)	−0.0011 (5)	0.0060 (5)	0.0024 (5)

### Geometric parameters (Å, º)

**Table:**

S1—C12	1.7717 (12)	C16—C17	1.3888 (19)
S1—C11	1.8011 (12)	C16—H16	0.9500
S2—C29	1.7729 (12)	C17—H17	0.9500
S2—C28	1.7944 (12)	C18—C28	1.5298 (16)
O1—C11	1.2021 (15)	C18—C25	1.5348 (15)
O2—C28	1.2033 (15)	C18—C19	1.5432 (15)
C1—C11	1.5284 (15)	C18—C26	1.5413 (15)
C1—C2	1.5360 (15)	C19—C20	1.5373 (16)
C1—C8	1.5413 (15)	C19—H19A	0.9900
C1—C9	1.5465 (15)	C19—H19B	0.9900
C2—C3	1.5357 (16)	C20—C21	1.5347 (17)
C2—H2A	0.9900	C20—C27	1.5356 (16)
C2—H2B	0.9900	C20—H20	1.0000
C3—C10	1.5337 (16)	C21—C22	1.5331 (17)
C3—C4	1.5359 (16)	C21—H21A	0.9900
C3—H3	1.0000	C21—H21B	0.9900
C4—C5	1.5349 (16)	C22—C26	1.5343 (17)
C4—H4A	0.9900	C22—C23	1.5322 (17)
C4—H4B	0.9900	C22—H22	1.0000
C5—C9	1.5330 (16)	C23—C24	1.5328 (17)
C5—C6	1.5343 (17)	C23—H23A	0.9900
C5—H5	1.0000	C23—H23B	0.9900
C6—C7	1.5339 (16)	C24—C27	1.5308 (16)
C6—H6A	0.9900	C24—C25	1.5368 (16)
C6—H6B	0.9900	C24—H24	1.0000
C7—C10	1.5349 (16)	C25—H25A	0.9900
C7—C8	1.5362 (16)	C25—H25B	0.9900
C7—H7	1.0000	C26—H26A	0.9900
C8—H8A	0.9900	C26—H26B	0.9900
C8—H8B	0.9900	C27—H27A	0.9900
C9—H9A	0.9900	C27—H27B	0.9900
C9—H9B	0.9900	C29—C30	1.3937 (18)
C10—H10A	0.9900	C29—C34	1.3926 (18)
C10—H10B	0.9900	C30—C31	1.3891 (18)
C12—C13	1.3894 (18)	C30—H30	0.9500
C12—C17	1.3917 (18)	C31—C32	1.386 (2)
C13—C14	1.3919 (18)	C31—H31	0.9500
C13—H13	0.9500	C32—C33	1.3902 (19)
C14—C15	1.381 (2)	C32—H32	0.9500
C14—H14	0.9500	C33—C34	1.3907 (18)
C15—C16	1.386 (2)	C33—H33	0.9500
C15—H15	0.9500	C34—H34	0.9500
			
C12—S1—C11	102.49 (6)	C12—C17—H17	120.4
C29—S2—C28	102.86 (6)	C28—C18—C25	114.10 (10)
C11—C1—C2	109.59 (9)	C28—C18—C19	108.16 (9)
C11—C1—C8	110.32 (9)	C25—C18—C19	109.05 (9)
C2—C1—C8	109.07 (9)	C28—C18—C26	107.72 (9)
C11—C1—C9	109.68 (9)	C25—C18—C26	108.69 (9)
C2—C1—C9	109.26 (9)	C19—C18—C26	109.00 (9)
C8—C1—C9	108.91 (9)	C20—C19—C18	109.60 (9)
C3—C2—C1	109.73 (9)	C20—C19—H19A	109.8
C3—C2—H2A	109.7	C18—C19—H19A	109.8
C1—C2—H2A	109.7	C20—C19—H19B	109.8
C3—C2—H2B	109.7	C18—C19—H19B	109.8
C1—C2—H2B	109.7	H19A—C19—H19B	108.2
H2A—C2—H2B	108.2	C21—C20—C27	109.38 (10)
C10—C3—C2	109.92 (10)	C21—C20—C19	109.20 (10)
C10—C3—C4	109.36 (10)	C27—C20—C19	110.03 (10)
C2—C3—C4	109.45 (10)	C21—C20—H20	109.4
C10—C3—H3	109.4	C27—C20—H20	109.4
C2—C3—H3	109.4	C19—C20—H20	109.4
C4—C3—H3	109.4	C22—C21—C20	109.54 (10)
C5—C4—C3	109.37 (9)	C22—C21—H21A	109.8
C5—C4—H4A	109.8	C20—C21—H21A	109.8
C3—C4—H4A	109.8	C22—C21—H21B	109.8
C5—C4—H4B	109.8	C20—C21—H21B	109.8
C3—C4—H4B	109.8	H21A—C21—H21B	108.2
H4A—C4—H4B	108.2	C21—C22—C26	109.73 (10)
C9—C5—C6	109.35 (9)	C21—C22—C23	109.30 (10)
C9—C5—C4	109.55 (10)	C26—C22—C23	109.58 (10)
C6—C5—C4	109.72 (10)	C21—C22—H22	109.4
C9—C5—H5	109.4	C26—C22—H22	109.4
C6—C5—H5	109.4	C23—C22—H22	109.4
C4—C5—H5	109.4	C24—C23—C22	109.24 (9)
C7—C6—C5	109.55 (9)	C24—C23—H23A	109.8
C7—C6—H6A	109.8	C22—C23—H23A	109.8
C5—C6—H6A	109.8	C24—C23—H23B	109.8
C7—C6—H6B	109.8	C22—C23—H23B	109.8
C5—C6—H6B	109.8	H23A—C23—H23B	108.3
H6A—C6—H6B	108.2	C27—C24—C23	109.71 (10)
C6—C7—C10	109.45 (9)	C27—C24—C25	109.04 (10)
C6—C7—C8	109.29 (9)	C23—C24—C25	109.83 (10)
C10—C7—C8	109.63 (10)	C27—C24—H24	109.4
C6—C7—H7	109.5	C23—C24—H24	109.4
C10—C7—H7	109.5	C25—C24—H24	109.4
C8—C7—H7	109.5	C18—C25—C24	110.28 (9)
C7—C8—C1	109.86 (9)	C18—C25—H25A	109.6
C7—C8—H8A	109.7	C24—C25—H25A	109.6
C1—C8—H8A	109.7	C18—C25—H25B	109.6
C7—C8—H8B	109.7	C24—C25—H25B	109.6
C1—C8—H8B	109.7	H25A—C25—H25B	108.1
H8A—C8—H8B	108.2	C22—C26—C18	109.94 (9)
C5—C9—C1	109.64 (9)	C22—C26—H26A	109.7
C5—C9—H9A	109.7	C18—C26—H26A	109.7
C1—C9—H9A	109.7	C22—C26—H26B	109.7
C5—C9—H9B	109.7	C18—C26—H26B	109.7
C1—C9—H9B	109.7	H26A—C26—H26B	108.2
H9A—C9—H9B	108.2	C24—C27—C20	109.31 (9)
C3—C10—C7	109.37 (9)	C24—C27—H27A	109.8
C3—C10—H10A	109.8	C20—C27—H27A	109.8
C7—C10—H10A	109.8	C24—C27—H27B	109.8
C3—C10—H10B	109.8	C20—C27—H27B	109.8
C7—C10—H10B	109.8	H27A—C27—H27B	108.3
H10A—C10—H10B	108.2	O2—C28—C18	123.17 (11)
O1—C11—C1	124.03 (11)	O2—C28—S2	122.56 (9)
O1—C11—S1	122.73 (9)	C18—C28—S2	114.26 (8)
C1—C11—S1	113.24 (8)	C30—C29—C34	120.65 (11)
C13—C12—C17	120.87 (12)	C30—C29—S2	117.47 (10)
C13—C12—S1	118.15 (10)	C34—C29—S2	121.66 (10)
C17—C12—S1	120.90 (10)	C31—C30—C29	119.34 (12)
C12—C13—C14	119.10 (13)	C31—C30—H30	120.3
C12—C13—H13	120.4	C29—C30—H30	120.3
C14—C13—H13	120.4	C32—C31—C30	120.40 (12)
C15—C14—C13	120.47 (13)	C32—C31—H31	119.8
C15—C14—H14	119.8	C30—C31—H31	119.8
C13—C14—H14	119.8	C31—C32—C33	120.01 (12)
C14—C15—C16	120.01 (12)	C31—C32—H32	120.0
C14—C15—H15	120.0	C33—C32—H32	120.0
C16—C15—H15	120.0	C34—C33—C32	120.26 (12)
C15—C16—C17	120.42 (13)	C34—C33—H33	119.9
C15—C16—H16	119.8	C32—C33—H33	119.9
C17—C16—H16	119.8	C33—C34—C29	119.33 (12)
C16—C17—C12	119.13 (13)	C33—C34—H34	120.3
C16—C17—H17	120.4	C29—C34—H34	120.3
			
C11—C1—C2—C3	−179.65 (9)	C28—C18—C19—C20	−176.73 (9)
C8—C1—C2—C3	59.48 (12)	C25—C18—C19—C20	58.66 (12)
C9—C1—C2—C3	−59.46 (12)	C26—C18—C19—C20	−59.87 (12)
C1—C2—C3—C10	−59.96 (12)	C18—C19—C20—C21	60.67 (12)
C1—C2—C3—C4	60.17 (12)	C18—C19—C20—C27	−59.40 (12)
C10—C3—C4—C5	60.16 (12)	C27—C20—C21—C22	60.01 (13)
C2—C3—C4—C5	−60.31 (12)	C19—C20—C21—C22	−60.45 (12)
C3—C4—C5—C9	60.34 (12)	C20—C21—C22—C26	59.90 (13)
C3—C4—C5—C6	−59.71 (12)	C20—C21—C22—C23	−60.30 (13)
C9—C5—C6—C7	−60.61 (12)	C21—C22—C23—C24	60.27 (12)
C4—C5—C6—C7	59.56 (12)	C26—C22—C23—C24	−60.02 (12)
C5—C6—C7—C10	−59.76 (12)	C22—C23—C24—C27	−60.40 (12)
C5—C6—C7—C8	60.31 (12)	C22—C23—C24—C25	59.43 (12)
C6—C7—C8—C1	−60.07 (12)	C28—C18—C25—C24	179.25 (9)
C10—C7—C8—C1	59.89 (12)	C19—C18—C25—C24	−59.70 (12)
C11—C1—C8—C7	179.96 (9)	C26—C18—C25—C24	59.03 (12)
C2—C1—C8—C7	−59.63 (12)	C27—C24—C25—C18	60.66 (12)
C9—C1—C8—C7	59.53 (12)	C23—C24—C25—C18	−59.58 (13)
C6—C5—C9—C1	60.35 (12)	C21—C22—C26—C18	−59.45 (13)
C4—C5—C9—C1	−59.92 (12)	C23—C22—C26—C18	60.57 (12)
C11—C1—C9—C5	179.52 (9)	C28—C18—C26—C22	176.34 (9)
C2—C1—C9—C5	59.38 (12)	C25—C18—C26—C22	−59.56 (12)
C8—C1—C9—C5	−59.66 (12)	C19—C18—C26—C22	59.20 (12)
C2—C3—C10—C7	59.66 (12)	C23—C24—C27—C20	60.11 (12)
C4—C3—C10—C7	−60.52 (12)	C25—C24—C27—C20	−60.21 (12)
C6—C7—C10—C3	60.32 (12)	C21—C20—C27—C24	−59.75 (13)
C8—C7—C10—C3	−59.55 (12)	C19—C20—C27—C24	60.20 (12)
C2—C1—C11—O1	−1.39 (16)	C25—C18—C28—O2	−173.14 (11)
C8—C1—C11—O1	118.72 (12)	C19—C18—C28—O2	65.32 (14)
C9—C1—C11—O1	−121.32 (12)	C26—C18—C28—O2	−52.37 (15)
C2—C1—C11—S1	178.25 (8)	C25—C18—C28—S2	6.81 (13)
C8—C1—C11—S1	−61.65 (10)	C19—C18—C28—S2	−114.73 (9)
C9—C1—C11—S1	58.32 (11)	C26—C18—C28—S2	127.58 (9)
C12—S1—C11—O1	1.36 (12)	C29—S2—C28—O2	4.68 (12)
C12—S1—C11—C1	−178.29 (8)	C29—S2—C28—C18	−175.27 (8)
C11—S1—C12—C13	−122.88 (10)	C28—S2—C29—C30	118.64 (10)
C11—S1—C12—C17	60.50 (11)	C28—S2—C29—C34	−66.73 (11)
C17—C12—C13—C14	0.09 (18)	C34—C29—C30—C31	−0.55 (19)
S1—C12—C13—C14	−176.54 (9)	S2—C29—C30—C31	174.15 (10)
C12—C13—C14—C15	−0.74 (19)	C29—C30—C31—C32	0.8 (2)
C13—C14—C15—C16	0.6 (2)	C30—C31—C32—C33	−0.1 (2)
C14—C15—C16—C17	0.3 (2)	C31—C32—C33—C34	−1.0 (2)
C15—C16—C17—C12	−0.92 (19)	C32—C33—C34—C29	1.24 (19)
C13—C12—C17—C16	0.74 (18)	C30—C29—C34—C33	−0.48 (19)
S1—C12—C17—C16	177.27 (10)	S2—C29—C34—C33	−174.95 (10)

### Hydrogen-bond geometry (Å, º)

Cg1 and Cg2 are the centroids of the C12–C17 and C29–C34 benzene rings, respectively.

**Table:**

*D*—H···*A*	*D*—H	H···*A*	*D*···*A*	*D*—H···*A*
C22—H22···*Cg*1^i^	1.00	2.81	3.6129 (13)	138
C34—H34···*Cg*1	0.95	2.73	3.4234 (14)	131
C15—H15···*Cg*2^ii^	0.95	2.86	3.6668 (16)	143

Symmetry codes: (i) *x*, *y*, *z*+1; (ii) *x*, −*y*−1/2, *z*−3/2.
